# Simultaneous cardio-cerebral embolization associated with atrial fibrillation: a case report

**DOI:** 10.1186/s12883-019-1388-1

**Published:** 2019-07-05

**Authors:** Soichiro Abe, Kanta Tanaka, Hiroshi Yamagami, Kazutaka Sonoda, Hiroya Hayashi, Shuichi Yoneda, Kazunori Toyoda, Masatoshi Koga

**Affiliations:** 1 Division of Stroke Care Unit, National Cerebral and Cardiovascular Center, 6-1 Kishibe Shinmachi, Suita, 564-8565 Japan; 2 Department of Neurology, National Cerebral and Cardiovascular Center, Suita, Japan; 3 Department of Cardiovascular Medicine, National Cerebral and Cardiovascular Center, Suita, Japan; 4 Department of Cerebrovascular Medicine, National Cerebral and Cardiovascular Center, Suita, Japan

**Keywords:** Cerebral embolization, Coronary embolization, Cardiocerebral infarction, Atrial fibrillation, Recanalization

## Abstract

**Background:**

Simultaneous cerebral and myocardial infarction is called cardiocerebral infarction (CCI), and is rarely encountered. Because of the narrow time window and complex pathophysiology, CCI is challenging to immediately diagnose and treat.

**Case presentation:**

A 73-year-old woman suddenly developed right hemiplegia and severe aphasia. Twelve-lead electrocardiography showed tachycardic atrial fibrillation without any significant ST-T change. Magnetic resonance imaging revealed a proximal middle cerebral artery occlusion. She was immediately treated with alteplase at the dosage approved for ischemic stroke followed by mechanical thrombectomy as bridging therapy, and complete recanalization was achieved. Aphasia improved and she began to complain of chest pain, and reported that she had experienced chest discomfort just prior to right limb weakness. Coronary angiography showed a partial filling defect in the right coronary artery with rapid and adequate distal flow, for which percutaneous coronary intervention was not required. Alteplase was suggested to have effectively resolved the coronary emboli. The occlusions of the cerebral and coronary arteries were assumed to have occurred nearly simultaneously and cardiogenic embolism due to atrial fibrillation was considered as the most likely etiology.

**Conclusions:**

As seen in the present case, CCI may benefit from immediate treatment with intravenous tissue plasminogen activator (IV-tPA). Although which of percutaneous coronary intervention or cerebral thrombectomy should be performed first remains unclear, we must decide whether to rescue the brain or heart first in each patient within a limited window of time. This dilemma has recently become evident in this era with mechanical thrombectomy strongly established as an effective intervention for acute ischemic stroke. Close cooperation between stroke physicians and cardiologists is becoming more important.

## Background

Simultaneous cerebral and myocardial infarction is called cardiocerebral infarction (CCI), and is rarely encountered [1]. Because of the narrow time window and the complex pathophysiology, CCI is challenging to immediately diagnose and treat. Here, we report the case of a patient with simultaneous cerebral and coronary embolization associated with atrial fibrillation, with consideration of the management strategy for CCI.

## Case presentation

**Fig. 1:**
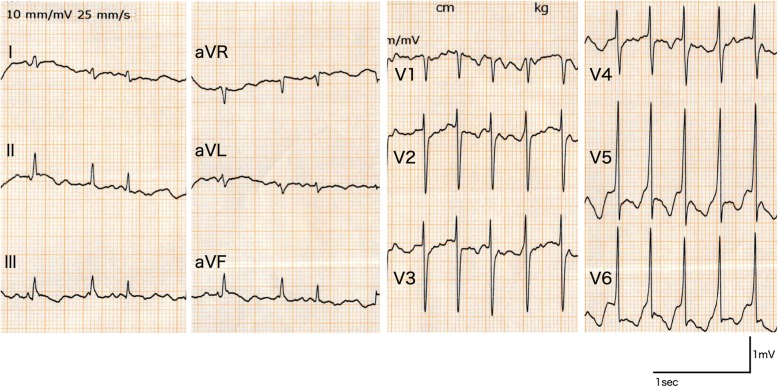
Twelve-lead electrocardiogram on admission showing tachycardic atrial fibrillation

A 73-year-old woman suddenly developed right hemiplegia and severe aphasia and was transported to our emergency service 47 min after onset. Her medical history included hypertension and paroxysmal atrial fibrillation. Anticoagulants have been discontinued because of a few episodes of falls although she had previously received oral anticoagulation. Blood pressure was 105/75 mmHg without any significant difference between right and left limbs. No cardiac murmurs were audible. Twelve-lead electrocardiography (ECG) showed tachycardic atrial fibrillation with a heart rate of 150 beats/min but no significant ST-T changes, although the baseline was undulating due to patient movement (Fig. 1). Glasgow Coma Scale score was 9 (E4V1M4). The patient was mute and could not follow any commands. She presented with right hemiplegia and showed left-ward conjugate eye deviation that could not be overcome with oculocephalic stimulation. The National Institutes of Health Stroke Scale score was 21. Blood glucose level was 189 mg/dL, serum creatinine level was 0.66 mg/dL, hemoglobin was 10.8 mg/dL, and platelet count was 16.9 × 10^4^/μL. No abnormalities were evident on chest roentgenogram.

**Fig. 2:**
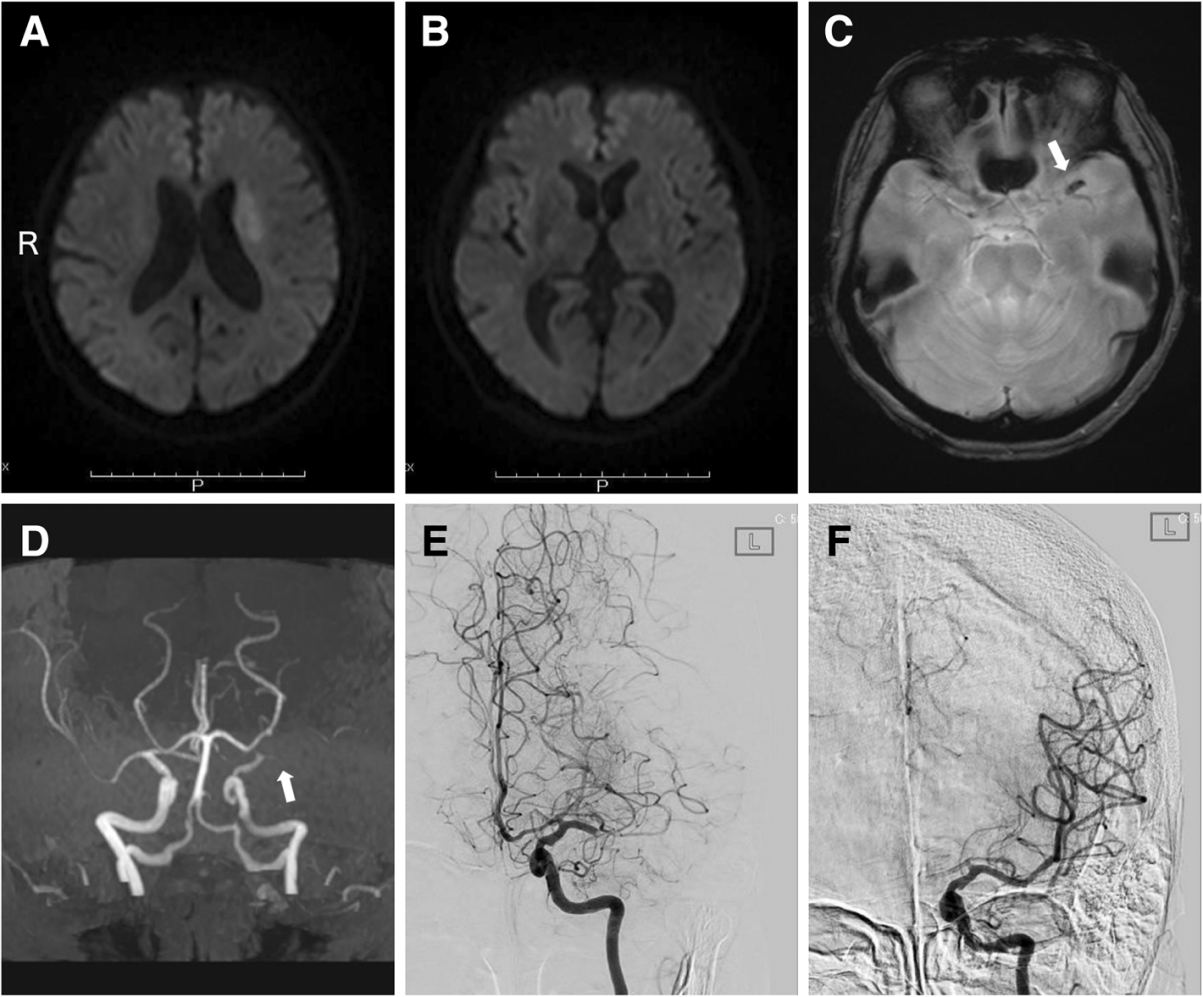
Diffusion-weighted magnetic resonance imaging showing acute infarction at the left corona radiata (**a**) and left basal ganglia (**b**). (**c**) T2*-weighted imaging showing a susceptibility vessel sign (white arrow) in the left middle cerebral artery. (**d**) Magnetic resonance angiography showing proximal occlusion (white arrow) of the left middle cerebral artery. (**e**) Anteroposterior view of a selective left internal carotid angiogram showing occlusion of the left middle cerebral artery. (**f**) Left internal carotid angiogram, anteroposterior view, showing complete recanalization of the left middle cerebral artery

We immediately performed magnetic resonance imaging following the stroke management protocol in our institute. Diffusion-weighted imaging showed hyperintense lesions at the left basal ganglia and corona radiata (Fig. 2a, b). A susceptibility vessel sign was seen in the proximal portion of the left middle cerebral artery (Fig. 2c). Magnetic resonance angiography revealed proximal occlusion of the left middle cerebral artery (Fig. 2d). Alteplase at a dose of 0.6 mg/kg (the dose approved in Japan) was administered 43 min after hospital arrival [2, 3], immediately followed by endovascular thrombectomy as bridging therapy [4]. After thrombectomy with a stent retriever (Solitaire 2, 4 × 20 mm; Medtronic, California, USA), complete recanalization was obtained 95 min after hospital arrival (Fig. 2e, f). Cardiac rhythm monitoring during the endovascular procedure did not show significant findings other than the tachycardic atrial fibrillation.

**Fig. 3:**
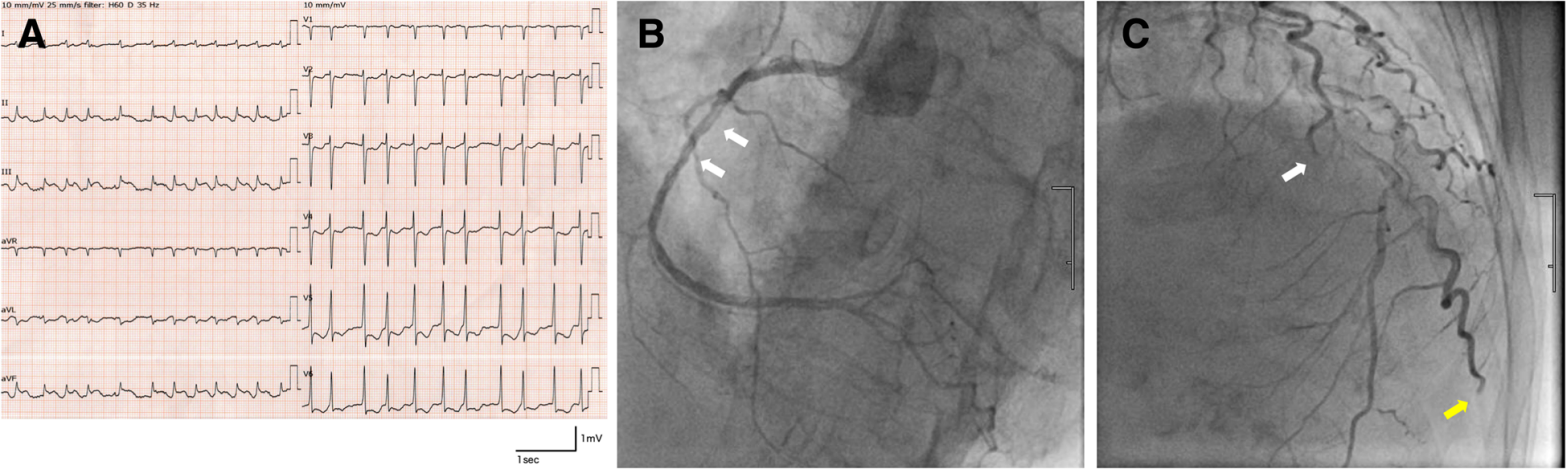
(**a**) Twelve-lead electrocardiogram after thrombectomy showing ST elevation at II, III, and aVF and ST depression at V2–6. (**b**) Left anterior oblique cranial view of coronary angiography showing filling defects (white arrows) in the right coronary artery. Distal flow is rapid and adequate. (**c**) Anteroposterior cranial view of coronary angiography showing distal occlusions of the left circumflex small branch (white arrow) and the diagonal branch (yellow arrow)

Aphasia and right hemiplegia improved immediately after completing mechanical thrombectomy and she began to complain of chest pain. A detailed medical history was elicited and revealed that she experienced chest discomfort just prior to the development of weakness in the right extremities. No symptoms suggestive of angina pectoris had been present prior to this episode of chest discomfort. Twelve-lead ECG showed ST elevation at II, III, and aVF and ST depression at V2–V6 (Fig. 3a). Transthoracic echocardiogram showed decreased motion of the posterior wall and interventricular septum, and serum troponins were elevated. Coronary angiography revealed a filling defect in the right coronary artery, but distal flow was rapid and adequate (Fig. 3b). Distal occlusions were identified in the left circumflex small branch and the diagonal branch (Fig. 3c). No stenotic lesions suggestive of atherosclerotic pathology were identified. Because we considered that myocardial perfusion was sufficient overall, percutaneous coronary intervention (PCI) was not performed.

The occlusions of the left middle cerebral artery and coronary arteries were assumed to have occurred nearly simultaneously, with cardiogenic embolism due to atrial fibrillation considered as the most likely etiology [5]. No deep vein thrombosis of the lower limbs was evident on ultrasonography. On day 5 after admission, oral rivaroxaban was started at a dose of 15 mg/day (the standard dose in Japan) [6]. Bisoprolol (0.625 mg/day) was initiated for rate-control purposes. The patient was discharged for rehabilitation on day 22. The modified Rankin Scale score at 3 months after stroke onset was 2. The patient experienced no recurrence of cerebral or myocardial infarction at 6 months after discharge.

## Discussion and conclusions

We have presented herein a case of atrial fibrillation-associated CCI in which nearly simultaneous embolization of the cerebral and coronary arteries occurred. The term CCI was coined by Omar et al. [1], and this rare condition has a reported frequency of 0.009–0.52% of ischemic strokes [7–9]. Recommendations or guidelines for the management of CCI remain lacking because of the rarity and variable pathophysiology of this clinical scenario. Among the 12 reported patients with detailed clinical information, no patient received oral anticoagulation prior to the event and 3 patients were newly diagnosed as having atrial fibrillation [1, 7, 10–14]. In our patient, appropriate anticoagulation might have prevented the event of CCI. Risk-benefit profile of oral anticoagulation for patients with tendency to fall should be elucidated further [15].

In our patient, chest discomfort was not initially detected due to the severe aphasia. The initial 12-lead ECG and subsequent monitoring of cardiac rhythm did not show any marked ST-T changes, as these would have been obscured by the tachycardic atrial fibrillation and the wandering baseline artifact, both of which are frequently encountered in emergency settings. Although evaluation of serum cardiac enzymes might have allowed earlier diagnosis of coronary embolization, routine monitoring of cardiac enzymes is not clearly recommended by major guidelines for acute ischemic stroke [16, 17]. In cases of cerebral embolism, it is important to constantly keep in mind the possibility of concomitant myocardial infarction, because the diagnosis of myocardial infarction could be delayed due to several factors, including impaired consciousness, aphasia, or reduced pain sensation.

In our case, cerebral embolism was treated first with intravenous tissue plasminogen activator (IV-tPA) and mechanical thrombectomy, followed by coronary angiography, which did not suggest any requirement for PCI. Coronary angiography suggested that alteplase at the dosage approved for ischemic stroke had resolved the coronary emboli. Because IV-tPA is effective for both cerebral and myocardial infarction, CCI is likely to benefit from immediate treatment with IV-tPA prior to endovascular therapy. Although the recommended dosages of IV-tPA differ between cerebral and myocardial ischemia, in 6 previously reported cases of CCI treated with IV-tPA therapy, the dosage of IV-tPA applied was that used for acute ischemic stroke [7, 10, 11] [12, 13]. When treating CCI patients with IV-tPA, close attention is required because the etiology of CCI includes aortic dissection [18, 19], which is a contraindication for IV-tPA. Furthermore, fatal complications such as cardiac rupture and tamponade may occur after IV-tPA [20, 21].

A dilemma exists regarding endovascular therapy: although the occluded cerebral and coronary arteries have to be recanalized as soon as possible in the case of CCI, prioritizing one therapy leads to delays in the other. In previous studies, four patients were treated with both PCI and cerebral thrombectomy [7, 14], with cerebral thrombectomy carried out first in 2 patients. One patient showed severe disturbance of consciousness due to the proximal posterior cerebral artery occlusion [3] and another patient had no abnormal findings on ECG in the emergency room [18]. Regarding the 2 patients in whom PCI was implemented first, circulatory dynamics were unstable. Although which of PCI or cerebral thrombectomy should be performed first remains unclear, a decision algorithm for acute management of CCI has been proposed [11], in which cerebral thrombectomy can be prioritized if the circulatory dynamics are stable. Nonetheless, we must decide to rescue the brain or heart first in each patient within a limited window of time. This dilemma has become evident in the present era with mechanical thrombectomy strongly established as an effective intervention for acute ischemic stroke. Close cooperation between stroke physicians and cardiologists is thus becoming more important.

In conclusion, simultaneous cerebral and coronary embolism is rare and its prompt and appropriate management is difficult. Coexistence of coronary embolization should be considered when planning the treatment strategy for acute cerebral embolism. Further cases need to be studied to resolve the dilemma inherent in the recanalization strategy for CCI.

## Data Availability

All the data generated or analyzed during this study are included in this article.

## Abbreviations

CCI: cardiocerebral infarction
ECG: electrocardiography
IV-tPA: intravenous tissue plasminogen activator
PCI: percutaneous coronary intervention
